# Topography of the Interfacial Shear Strength and the Mean Intrinsic Tensile Strength of Hemp Fibers as a Reinforcement of Polypropylene

**DOI:** 10.3390/ma13041012

**Published:** 2020-02-24

**Authors:** David Hernández-Díaz, Ricardo Villar-Ribera, Fernando Julián, Quim Tarrés, Francesc X. Espinach, Marc Delgado-Aguilar

**Affiliations:** 1 Serra Húnter Programme, Department of Graphical Engineering and Design, Universitat Politècnica de Catalunya, Campus Terrassa, 08222 Terrassa, Spain; david.hernandez-diaz@upc.edu; 2 Department of Graphical Engineering and Design, Universitat Politècnica de Catalunya, Campus Manresa, 08242 Manresa, Spain; villar@ege.upc.edu; 3 Design, Development and Product Innovation, Department of Organization, Business, University of Girona, 17003 Girona, Spain; fernando.julian@udg.edu; 4 LEPAMAP Group, Department of Chemical Engineering, University of Girona, 17003 Girona, Spain; joaquimagusti.tarres@udg.edu (Q.T.); m.delgado@udg.edu (M.D.-A.)

**Keywords:** micromechanics, tensile strength, interfacial shear strength, hemp, composites

## Abstract

The strength of the interphase between the reinforcements and the matrix has a major role in the mechanical properties of natural fiber reinforced polyolefin composites. The creation of strong interphases is hindered by the hydrophobic and hydrophilic natures of the matrix and the reinforcements, respectively. Adding coupling agents has been a common strategy to solve this problem. Nonetheless, a correct dosage of such coupling agents is important to, on the one hand guarantee strong interphases and high tensile strengths, and on the other hand ensure a full exploitation of the strengthening capabilities of the reinforcements. The paper proposes using topographic profile techniques to represent the effect of reinforcement and coupling agent contents of the strength of the interphase and the exploitation of the reinforcements. This representation allowed identifying the areas that are more or less sensitive to coupling agent content. The research also helped by finding that an excess of coupling agent had less impact than a lack of this component.

## 1. Introduction

The tensile strength of short fiber semi-aligned composites is heavily impacted by the chemical nature and the mechanical properties of the phases, the strength of the interphase between the matrix and the reinforcements, the morphology of the reinforcements and the mean orientation and dispersion of the reinforcements [1,2,3]. Some of such parameters, like the nature of the phases, its mechanical properties or the morphology of the reinforcements are or known or experimentally measurable. Nonetheless, the strength of the interphase, the dispersion or the mean orientation of the fibers against the applied loads are not so easy to measure [4].

The nature of the phases implies a good or a bad compatibility and wettability. In the case of natural fiber reinforced polyolefin, both phases present notable differences. While natural fibers are hydrophilic, polyolefin is hydrophobic, leading to weak interphases and the presence of voids in the interphase. This problem has been widely discussed in the literature, and some solutions based on fiber treatments, surface treatments or the use of coupling agents have been proposed [5,6,7,8,9,10,11]. Mechanical, thermo-mechanical or chemo-thermo-mechanical treatments have been used to modify the chemical composition of the fiber surfaces, reducing the presence of lignin and extractives and increasing the presence of cellulose and hemicelluloses [12,13,14,15]. Furthermore, the use of maleic anhydride-grafted polyolefin as a coupling agent has provided a solution to obtain strong interphases [16,17,18,19]. However, the percentage of coupling agent against reinforcement content impacts the strength of the interphase and must be precisely dosed [18]. The literature shows how the tensile strength of the composites increases with the percentage of coupling agent up to a point. Then, higher coupling agent contents led to a decrease of such composite tensile strengths [18]. These decreases can be attributed to coupling agent self-bonding [12,13]. The literature fully discussed the impact of the coupling agents and the chemical reactions, but to the best knowledge of the authors, this involved using a discrete approach. 

The mechanical properties of the phases have also a big impact on the mechanical properties of the composite. While the tensile strength of the matrix can be obtained by performing a standard tensile test, the intrinsic tensile strength of natural reinforcements is not so easy to obtain. On the one hand, natural fibers show a high standard deviation in such values, due to its nature and the impact of meteorology, origin, etc. [20,21]. On the other hand, some natural fibers are presented in the shape of strands and can be tensile tested, but other natural fibers, due to its length can prove more difficult to test, although there are ingenious test procedures that allow its testing [3,22,23,24]. Furthermore, it is known that the morphology of the fibers changes during composite preparation and its length and diameter can change due to attrition phenomena or lumen collapse [25]. Thus, some authors defend the use of micromechanics models to measure the intrinsic tensile strength of the reinforcements [25,26,27,28]. These micromechanics models can deliver values different to those measured directly from the reinforcement, and some authors identify a possible link between the intrinsic tensile strength of a reinforcement in a composite and the chemical nature of the matrix [29,30,31]. The literature on the measurement of the intrinsic tensile strength of different fibers is wide, but to the best knowledge of the authors, its continuous behavior against fiber volume fraction and coupling agent content has not been addressed.

Short fiber semi-aligned reinforced composites show three main areas of reinforcement orientation [32,33]. In the skin area, the closest to the injection mold walls, the orientation of the fibers is either random or in the direction of the flow, depending on the geometry of the mold. Below the skin, there is the shell area, where the reinforcements are heavily oriented with the material flow. The inner area, called the core, orients the fibers transverse to the flow. The literature shows how it is possible to obtain an orientation factor (*χ*_1_) and how this factor is impacted by the injection mold geometry and the processing parameters [13,34,35]. The authors have found that orientation factor usually stands between 0.25 and 0.35 when producing standard specimens in their lab equipment, despite the matrix or the reinforcement natures and percentages [1,2,23,27]. Thus, this orientation factor is used to verify the results. 

The dispersion of the fibers in the matrix is difficult to assess. Some authors use scanning electron microscope (SEM) micrographs to discuss the proper dispersion of the reinforcements [36]. Nonetheless, other authors consider this method to be too descriptive and prefer using the behavior of some mechanical properties against the reinforcement content as clues of a good dispersion. This authors link a linear increase of the tensile strength of the composites against reinforcement content as a clue of a strong interphase and a proper fiber dispersion [23,27]. Likewise, a linear increase of the Young’s modulus of the composites against reinforcement content is seen as a clue of a proper fiber dispersion [14,37].

Glass fiber reinforced polypropylene composites are commodity materials for automotive and consumer goods industries. The literature shows multiple examples where the mechanical properties of these materials were used as a goal [2,23,27]. In these papers, the researchers propose replacing glass fiber with other natural fibers, in order to obtain similar mechanical properties [4,30,38,39]. Thus, polypropylene has been widely studied as a material prone to be reinforced with natural fibers, obtaining mechanical properties comparable to commercial products [40]. On the other hand, the tensile strength and the stiffness of hemp strand reinforced polypropylene composites is present in the literature [5,25,35]. These papers describe the mechanical properties of such composites and also study its micromechanics. In an interesting paper about the tensile strength, the authors found strong interphases but did not discuss the evolution of the properties against coupling agent contents [25]. In another paper, the authors discussed a linear evolution they had found of the Young’s modulus against reinforcement contents, as well as the proper dispersion of the reinforcements [35]. 

In this work, composite materials based on polypropylene (PP) reinforced with hemp strands were obtained and tensile tested. The reinforcement contents ranged from 20% to 50% *w*/*w*. In order to obtain strong interphases, coupling agent percentages ranging from 2% to 8% *w*/*w* were added to the composite formulations. The composites were mold injected to obtain standard specimens that were tensile tested. The macro-properties and its behavior against reinforcement and coupling agent percentages were presented and discussed. Next, a micromechanics model was used to obtain the interfacial shear strength, an orientation factor and the intrinsic tensile strength of the reinforcements. The authors used a method based on topographic maps to present the evolution of the interfacial shear strength and the intrinsic strength of the fibers against reinforcement and coupling agent percentages. This novel presentation informs the researchers on the sensibility of such micromechanics properties against the composite formulation. Using a continuous representation method increases the information in the hands of the researchers in order to obtain better interphases. The research also stablishes the impact of coupling agent contents on the tensile properties of hemp strands reinforced polypropylene composites. 

## 2. Materials and Methods 

### 2.1. Materials

Agrofibra S.L. (Puigreig, Spain) kindly provided the untreated hemp strands (HS). These strands showed initial lengths of 200 to 300 mm. 

A polypropylene (PP) ISPLEN^®^ 090 by REPSOL YPF (Tarragona, Spain) was used as a polymeric matrix. This polymer shows a density of 0.906 g/cm^3^ and a high fluidity. 

In order to obtain stronger interphases, a coupling agent EPOLENE^®^ G3015 by Eastman Chemical (Middelburg, Netherlands) was added in different percentages to the composites. This coupling agent is a modified maleic anhydride-grafted polypropylene (MAPP) with an Acid Number of 12.0 to 18.0 (mg-KOH).

A Xylene, by Sigma-Aldrich Chemie GmbH (Taufkirchen, Germany) was used to solve the polypropylene and recover the reinforcements.

### 2.2. Hemp Strands Treatment

The initial length of the hemp strands did not allow its use as reinforcement for a mold injected composite. Hence, these strands were cut to 10 mm length in a knife mill by Agrimsa (Sant Adrià del Besos, Spain). The initial raw material was a mix of hemp strands and hemp core fibers (25% *w*/*w*) [41]. The separation took place in a flotation cell. The recovered strands were dried in an oved at 80 °C for a minimum of 24 h.

### 2.3. Composites Formulation and Preparation

Composite materials with 20%, 30%, 40% and 50% *w*/*w* reinforcement contents were formulated and prepared. In order to obtain strong interphases, different percentages of MAPP were added to such composite materials. Afterwards, 2%, 4%, 6% and 8% MAPP contents (with respect to the reinforcement weight content) were tested. Hemp strands (HS), PP and MAPP were mixed in a roll mixer by IQAP LAB, S.L. (Les Masies de Roda, Spain). The process was carried out for an average of 10 min to ensure there was a proper dispersion of the HS. The machine was operated at 180 ± 5 °C. The obtained blends were pelletized in a knife mill by Agrimsa (Girona Spain) and dried at 80 °C for at least 24 h.

The notation used to label the composites was: xHSyMAPP, where x and y are the HS and MAPP wt % contents, respectively.

### 2.4. Sample Obtaining and Testing

The test specimens were mold injected in a Meteor 40 injection machine, by Mateu and Sole (Barcelona, Spain), following ASTM D638 standard [42]. The injection temperature profile was 170–185–200 °C, corresponding to the three heating areas of the equipment. First and second pressures were 120 and 37.5 kgf/cm^2^, respectively. At least 10 specimens for every composite formulation were obtained. 

The dog bone specimens were stored in a conditioning chamber at 23 °C and 50% relative humidity during at least 48 h. Then, the specimens were tensile tested in a Universal testing machine (instron TM 1122) by Instron (Norwood, MA, USA) fitted with a 5 kN load cell. The tests agreed with ASTM D790 [43]. At least 5 samples for every composite formulation were tested.

### 2.5. Extraction of the Hemp Strands from the Composite

It is accepted that the reinforcements are submitted to morphologic changes during the composite preparation. In order to evaluate such changes and use the appropriate morphologic data during the micromechanics modeling, the reinforcements were recovered by matrix solubilization in a Soxhlet apparatus (Merck Life Science S.L.U., Madrid, Spain). Small pieces of the specimens were cut and placed inside the apparatus in a cellulose filter. The process took 24 h. Then, the recovered HS were rinsed with acetone and washed with distilled water. Finally, the HS were dried for 24 h at 105 °C.

### 2.6. Morphology of the Hemp Strands

Fiber length distributions, diameters, and the percentage of fines were measured in a MORFI Compact analyzer by using Techpap (Grenoble, France). The equipment measured between 25,000 and 30,000 fibers. Four samples of each type of fiber were analyzed discriminating between fibers and fines. All the elements with length lower than 0.1 mm were considered to be fines.

## 3. Micromechanics

### 3.1. Modified Rule of Mixtures

A modified rule of mixtures was initially formulated for the Young’s modulus of semi oriented short fiber reinforced composites [44]. Nonetheless, the literature shows its usefulness in the case of the tensile strength [25,45,46]. The equation models the tensile strength of a composite as a sum of the contributions of the reinforcement and the matrix:

(1) $$\sigma_{t}^{C}=f_{c}\sigma_{t}^{F}V^{F}+\left(1-V^{F}\right)\sigma_{t}^{m*}.$$

In the equation, *σ_t_^C^*, *σ_t_^F^* and σ_t_^m*^ refer to the tensile strength of the composite, the intrinsic tensile strength of the reinforcement, and the contribution of the matrix at the strain at break of the composite, respectively. The reinforcement volume fraction is referred as *V^F^*, and *f_c_* is a coupling factor that equalizes the contribution of the reinforcements accounting for the strength of the interphase, the length of the reinforcements and its mean orientation. The tensile strength of the composite and the contribution of the matrix can be obtained experimentally, but the intrinsic tensile strength of short fibers cannot be determined in this way [4]. Thus, the equation shows two unknowns: *σ_t_^F^* and *f_c_*.

### 3.2. Kelly and Tyson Modified Equation

Kelly and Tyson presented a model to deal with aligned short fiber reinforced composites [47]. This model is based on a rule of mixtures and distinguishes between subcritical and supercritical fibers, as defined by the shear lag model [26]. The model defines a critical length (*L_C_*) as the minimum fiber length needed to transfer enough load through the interphase to break such a fiber.

(2) $$L_{C}=\frac{\sigma_{t}^{F}D^{F}}{2\tau }$$

In the equation, *D^F^* is the mean width of the reinforcements and *τ* the interfacial shear strength (MPa). The interfacial shear strength measures the ability of the interphase to transfer loads from the matrix to the reinforcement.

Furthermore, the Kelly and Tyson equation was modified to deal with semi-aligned reinforcements by adding an orientation factor (*χ*_1_):

(3) $$\sigma_{t}^{C}=\chi_{1}\left(X+Y\right)+Z.$$

In the equation, *X* and *Y* are the contributions of the subcritical and supercritical reinforcements, respectively, and *Z* is the contribution of the matrix.

Kelly and Tyson expanded the contributions as:

(4) $$X=\sum_{i}^{L_{i}<L_{c}}\frac{\tau L_{i}V_{i}^{F}}{D^{F}}$$

(5) $$Y=\sum_{j}^{L_{j}>L_{c}}\sigma_{t}^{F}V_{j}^{F}\left(1-\frac{\sigma_{t}^{F}V_{j}^{F}}{4\tau L_{j}}\right)$$

(6) $$Z=\left(1-V^{F}\right)\sigma_{t}^{m*}.$$

The fiber length distribution is needed to solve the equation. Similarly to for the modified rule of mixtures, the Kelly and Tyson equation shows too much unknowns to allow its solution. The literature shows methods to measure *σ_t_^F^*, *τ* and *χ*_1_, but in the case of short natural fiber need special equipment or are not fully reliable. Some of the authors measured the intrinsic strength of cotton and abaca strands, and found some deviations with respect to computed values [23,48]. In fact, some authors discuss the differences between the properties of the reinforcements outside and inside a composite [31].

### 3.3. Bowyer and Bader Method

Bowyer and Bader proposed a method to solve the Kelly and Tyson equation [49]. The solution considers that all the phases and the composite sustain the same strains (*ε_t_^C^* = *ε_t_^F^* = *ε_t_^m^*). Then, it attends to the relation between stress, strain and Young’s modulus, *σ_t_^F^* = *E_t_^F^ ε_t_^F^* = *E_t_^F^ ε_t_^C^*. The method evaluates the contributions of the phases at two points below the yield strength of the composite, and uses numerical methods to find *τ* and *χ*_1_.

Then, the only remaining unknown is the intrinsic tensile strength of the reinforcement and Equation (3) can be used for these purposes.

### 3.4. Hirsch Model

Hirsch equation was used to obtain the intrinsic Young’s modulus of the reinforcement. Hirsch equation is a linear combination of the Voigt and Reuss models [50]:

(7) $$E_{t}^{C}=\beta \left(E_{t}^{F}V^{F}+E_{t}^{m}\left(1-V^{F}\right)\right)+\left(1-\beta \right)\frac{E_{t}^{F}E_{t}^{m}}{E_{t}^{m}V^{F}+E_{t}^{F}\left(1-V^{F}\right)}$$

where *E_t_^C^*, *E_t_^F^* and *E_t_^m^* are the Young modulus of the composite, fiber and matrix, respectively. A parameter related with the stress transfer between the fiber and the matrix *β*, with a value of 0.4 has been used. The literature agrees that such value accurately describes the experimental behavior for natural short fiber composites (Kalaprasad et al., 1997).

The first part of the equation counts the contribution of fibers totally aligned with the loads, while the second half accounts for fibers that are perpendicular to such loads.

## 4. Results and Discussion

One of the main objectives of the research is to identify the impact of coupling agent contents on the tensile properties of the composites. With this objective, composite materials with reinforcement contents ranging from 20 to 50 wt % were prepared. Five batches of the composites, at 0 to 8 wt % MAPP contents (with respect to HS content) were prepared. Table 1 shows the experimental values obtained before the standard specimens were tensile tested.

**Figure 1:**
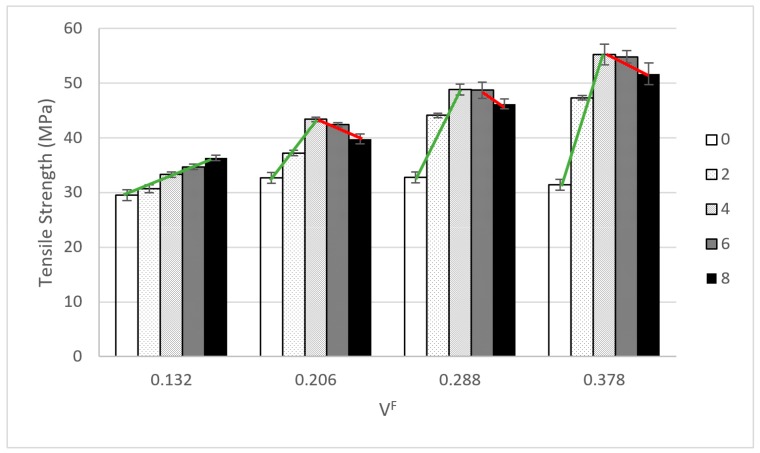
Evolution of the tensile strength of hemp strand reinforced polypropylene composites against coupling agent contents.

Regarding the tensile strength, it is apparent that coupling agents have a clear impact. The tensile strength of coupled composites is always higher that the uncoupled ones, despite the percentage of MAPP. Nonetheless, while the tensile strength of the composites increased with the coupling agent contents, in all the cases a maximum value can be identified, and only in the case of the composites at 20 wt % HS contents coincide with the highest MAPP content (Figure 1).

The composites with a 20 wt % of HS showed a linear evolution of its tensile strength against MAPP content, and the maximum was identified at 8 wt % of MAPP. Nonetheless, the rest of composites showed an initial fast increase of the tensile strength against MAPP content until a point where the tensile strength started to decrease. In these cases, the maximum tensile strength was identified for the 4 and 6 wt % MAPP contents. Notwithstanding, the differences between these values are not statistically relevant at a 95% confidence rate. Similar behaviors were observed in other natural fiber reinforced composites [39,51]. The slope of the regression lines increased with the HS contents. This can be related with the increasing amount of possible interactions between reinforcement and matrix when the percentage of reinforcement is increased. Thus, the yield of the coupling agent increased with the amount of reinforcement. Nonetheless, there is a point where the tensile strength starts to decrease. The literature blames this behavior on a self-entanglement of MAPP chains that hinders the creation of interactions with the OH groups of the reinforcements [4]. A preliminary analysis situates the optimum amount of MAPP, to maximize the tensile strength of the composites, between 4 and 6 wt % for the composites adding 30 to 50 wt % of HS. The composites adding a 20 wt % of HS showed a maximum value for an 8 wt % MAPP content. The tensile strength of a composite is heavily linked to the strength of its interphase. Thus, it can be inferred that the addition of coupling agents had a relevant role in the increase of the strength of such interphase. Nonetheless, a micromechanics analysis is needed to measure the strength of the interphase and the role of other parameters like the morphology of the fibers or its mean orientation [25,26]. It must be stressed that the percentages of coupling agents were stablished against reinforcement content. Thus, in absolute value, the composites with higher reinforcement contents added higher MAPP contents.

**Figure 2:**
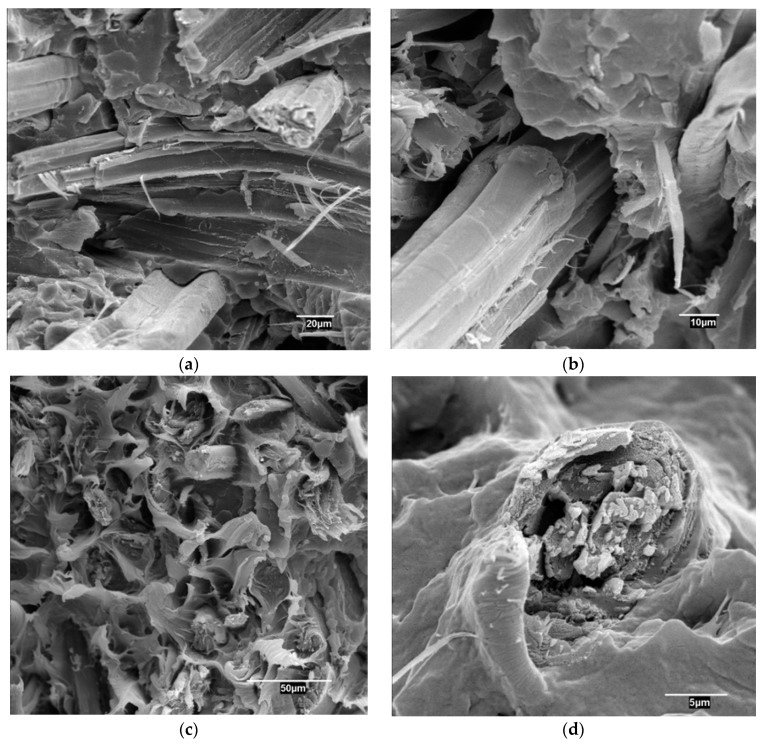
Scanning electron microscope (SEM) micrographs of the sections broken under impact for PP composites reinforced with 40 wt % of HS; (**a**,**b**): uncoupled composites, (**c**,**d**): composites adding 4 wt % of MAPP.

Figure 2 shows SEM micrographs for coupled and uncoupled composites.

Uncoupled composites (Figure 2a,b) show noticeable voids all around the interphase between the fibers and the matrix. These voids are a manifestation of the low adhesion between both phases. Moreover, the fibers are cut over the matrix surface, indicating slip out mechanisms. Pulled out fibers show clean surfaces without any trace of matrix. Coupled composites (Figure 2c,d) show a compact interphase, without voids. Figure 2d shows a fiber that is totally covered by the matrix and broken in the same plane as the matrix. These are indications of the presence of a strong interphase.

Table 1 shows the low impact of coupling agents on the Young’s moduli of the composites. The value of such moduli changed with the reinforcement contents but not with the MAPP percentages. The differences between the moduli of the composites with the same HS contents and different MAPP percentages were not statistically relevant, at a 95% confidence rate. This was expected, as the strength of the interphase is known to have little impact on the Young’s modulus of a composite [38,51].

The evolution of the strain at break of the composites increases in parallel with its tensile strength as a direct consequence of the relation between Young’s modulus (*E_t_*), tensile strength (*σ_t_*) and deformation (*ε_t_*): $\sigma_{t}=\varepsilon_{t}\cdot E_{t}$ (Table 1).

**Figure 3:**
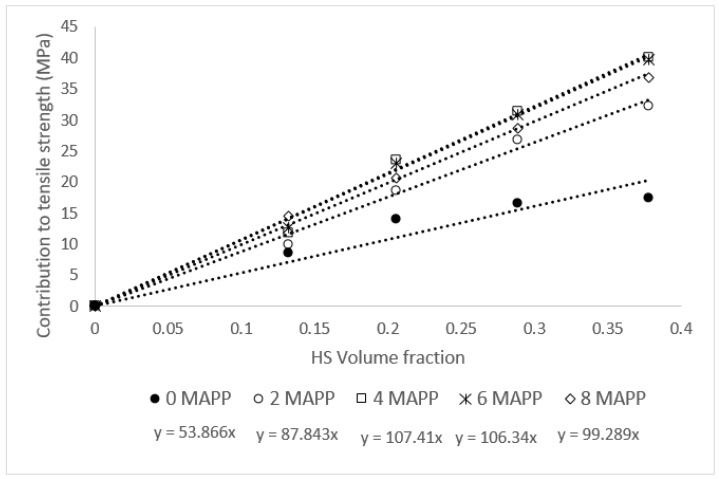
Contribution of the reinforcements to the tensile strength of the composite. Effect of the percentages of a coupling agent.

In the proposed rule of mixtures (Equation (1)), the contribution of the reinforcements to the tensile strength of the matrix equals *f_c_·σ_t_^F^*. If this value is plotted against the fiber volume fraction, then it returns a line (Figure 3). The slope of such lines is a measurement of the contribution of the reinforcements.

In the literature, the slope of these regression lines is referred as fiber tensile strength factor (*FTSF*) [23,45,48]. Apparently, the reinforcements at 4 wt % MAPP content returned the highest contributions. Nonetheless, the composites with 6 wt % contents returned similar values, which were only 1% lower. Higher MAPP contents decreased noticeably the contribution of the fibers, possibly due to self-bonding between the maleic acid parts of the coupling agent, reducing the number of interactions with the fiber surface. In the case of the uncoupled materials, the contribution of the reinforcements is not linear and shows an asymptotic behavior to values around 17 MPa for reinforcement contents higher than 30 wt%. Adding a 2 wt % of MAPP changes drastically the contribution of the fibers increasing the *FTSF* from 53.9 to 87.8. At the exception of the composites at 20 wt % reinforcement content, the highest reinforcement contributions were obtained with 4 wt % MAPP contents. In the case of the composites at 20 wt % reinforcement content, an 8 wt % of MAPP was necessary to obtain the highest fiber contribution.

The contribution of the fibers is a result of the strength of the interphase and the intrinsic tensile strength of the reinforcement, as well as the mean orientation of the reinforcements against the loads. A micromechanics analysis was carried out in order to verify the impact of the mentioned parameters on the contribution of the fibers. Table 2 shows the results obtained after solving the Kelly and Tyson modified equation (Equation (3)).

**Figure 4:**
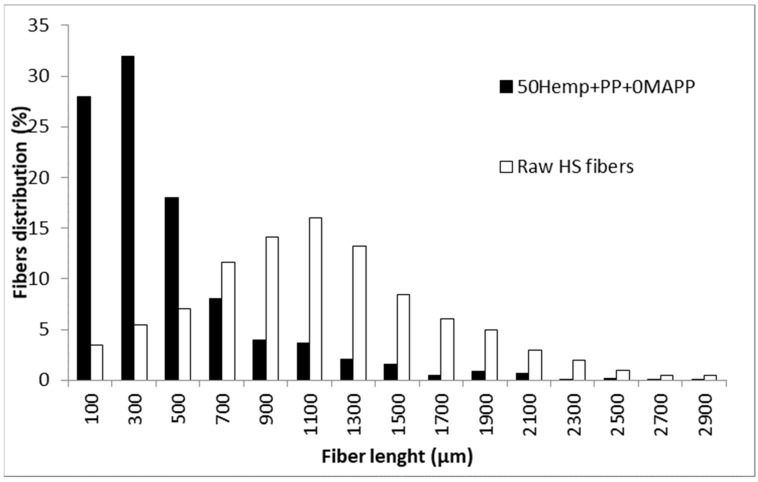
Fiber length distributions for the raw HS and the fibers extracted from an uncoupled composite with 50 wt % HS contents.

The table shows the impact of reinforcement contents of the mean length (*L^F^*) of the reinforcements. These lengths decreased from 670 µm to 371 µm for 20 to 50 wt % reinforcement contents. The literature attributes such fiber shortening to attrition phenomena that increases with the reinforcement contents [52]. This is of importance because the contribution of the subcritical fibers (Equation (4)) is usually lower than the contribution of supercritical fibers (Equation (5)). Thus, fiber shortening decreases the exploitation of the strengthening capabilities of the reinforcements. The coupling agent contents had a visible but minor impact on the mean length of the reinforcements. The length, at the exception of the composites at 40 wt % reinforcement contents, tend to decrease with the percentage of MAPP. Nonetheless, an ANOVA analysis can be necessary to verify if the observed differences are statistically relevant. Figure 4 shows the fiber length distribution after recovering the reinforcements from an uncoupled composite and the distribution for the raw HS.

The fibers experimented a noticeable shortening due to the attrition during mixing. The presence of long fibers was drastically reduced, and the shape of the distribution changed from a normal distribution to a distribution with a higher presence of short fibers. Most of the recovered fibers (78%) showed lengths below 700 µm. This is important because depending on the critical length, the number of supercritical fibers can decrease drastically, and its contribution can be compromised. The diameter of the fibers was less affected by the mixing and had a mean value of 30.75 µm.

**Figure 5:**
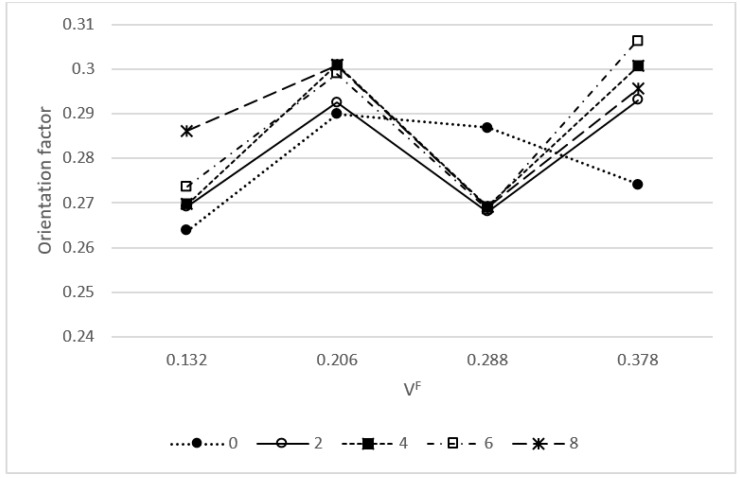
Evolution of the orientation factor against reinforcement contents.

The second parameter is the orientation factor (*χ*_1_). This factor is affected by the geometry of the injection mold and the injection molding parameters [27]. Other natural fiber reinforced polyolefin, mold injected with the same mold and equipment, returned orientation factors in the range from 0.25 to 0.35 [1,23]. The obtained values are inside the expected range and the other values were considered valid. Figure 5 shows the behavior of the orientation factor against reinforcement contents.

The obtained values evolved inside the 0.25 to 0.35 expected range. Coupled composites showed similar behaviors with values changing from 0.27 to 0.30. The ups and downs were more related with the solution of the equation than with the real mean orientation of the fibers. Anyhow, the orientation factor can be translated to mean orientation angles (*α*) by applying *χ*_1_ = cos^4^(*α*) [25]. The obtained mean orientation angles are in the range from 43.7° to 42.3° for orientation factors with 0.27 and 0.3 values, respectively.

The interfacial shear strength (*τ*) informs on the strength of the interphase between the reinforcements and the matrix. The literature accepts that interphases with values in the range defined by Tresca ($\tau =\sigma_{t}^{m}/2$) and von Mises ($\tau =\sigma_{t}^{m}/\sqrt{3}$) criteria can be accepted as strong ones [18]. Thus, interfacial shear strengths in the range from 13.8 to 15.9 MPa can be considered strong. Table 2 shows the impact of the percentage of MAPP on such value. The presence of MAPP, up to certain percentages increases the value of *τ* noticeably. Nonetheless, the percentage of increase, the maximum and the inflection points vary also with the reinforcement percentage. Figure 5 shows the same data presented as a topographic profile.

**Figure 6:**
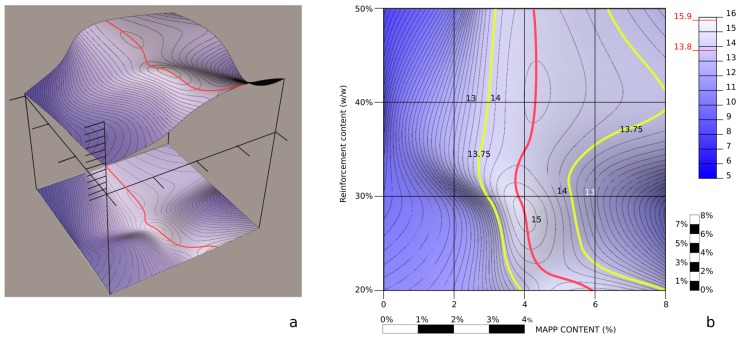
Topographic profile of the interfacial shear strength against coupling agent and reinforcement contents; (**a**): 3d process used to obtain iso-*τ* lines, (**b**): 2d view of the topographic profile.

The profile was obtained from a matrix of the values of *τ* against MAPP and reinforcement contents. These values were input as 3D points in a surface modeling software (Rhinoceros 3D). Then a surface passing through the points was obtained (Figure 6a). Planes with heights coinciding with integer *τ* values and then ¼, ½ and ¾ values intersected the surface to obtain the iso-*τ* profiles. A color map was projected to the surface to paint different heights with different colors. Afterwardsn the side view was projected to obtain a curve presenting the maximum height (red line). Finally, the information was projected to a 2D view (Figure 6b). Yellow lines present the Tresca criteria.

Figure 6b shows how the maximum interfacial shear strengths were obtained with MAPP percentages in the range from 4 to 6 wt %. It can be observed by the separation of the iso-*τ* how an excess of MAPP has less impact than low percentages of a coupling agent. The percentage with less latitude to obtain strong interphases is 30 wt % of reinforcement, coinciding with the area where the yellow lines are closer. Higher percentages of reinforcement allow a wider range of MAPP content without losing the strength of the interphase. In the case of the composites reinforced with a 20 wt %, the maximum interfacial shear strength was obtained for a 6 wt % MAPP content. This increase on the percentage of the coupling agent can be due to the highest mean length of the reinforcements (Table 2). Possibly in this case, the percentage of supercritical fibers was higher, and the relative area was as well. Nonetheless, adding a 4 wt % of coupling agent delivered a strong interphase.

**Figure 7:**
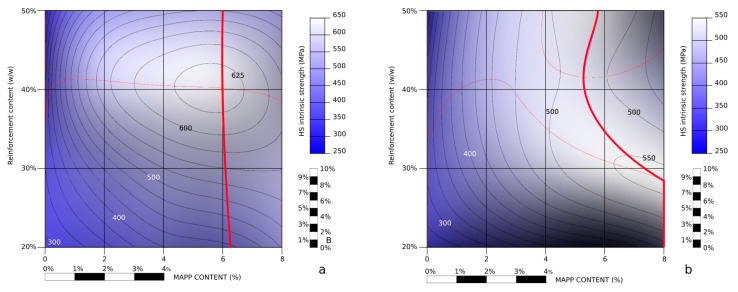
Topographic profile of the intrinsic tensile strength of the reinforcement against coupling agent and reinforcement contents; (**a**): maintaining the values obtained for *χ*_1_, (**b**): imposing a constant 0.3 orientation angle.

Table 2 shows the intrinsic tensile strength of the reinforcements. This strength had a similar behavior than the tensile strength of the composite and its interfacial shear strength. Being the same reinforcement, despite the presence of the coupling agent, it may be thought that the intrinsic tensile strength must be invariable. Nonetheless, the literature agrees that there are differences between the intrinsic tensile strength of a fiber measured independently or computed by micromechanics [31]. The obtained value must be seen as a measure of the use of the strengthening capabilities of the reinforcements. Figure 7 presents a topographic profile of the intrinsic tensile strength of the reinforcement against MAPP and reinforcement contents. The figures were with the same methodology used to obtain Figure 6.

Figure 7a shows how at a fixed MAPP percentage, adding reinforcement contents increase the obtained intrinsic tensile strength, up to 40 wt % contents, were the intrinsic tensile strength shows a tendency to descend. A local maximum value was found for a 40 wt % reinforcement content and 6 wt % MAPP content. This percentage of coupling agent does not coincide with the maximum tensile strength of the composite. Nonetheless, the values obtained with 4 and 6 wt % of MAPP were almost the same. On the other hand, the intrinsic tensile strength of the fibers inside composites with 4 wt % MAPP contents or higher, tend to keep almost constant. The zone between 0 and 2 wt % MAPP contents show almost vertical lines, marking the area where the intrinsic tensile strength of the reinforcement increases most notably. The decrease of the exploitation of the strengthening capabilities of the reinforcements at high fiber contents can be due to a more difficult dispersion and the creation of fiber bundles or fiber to fiber contacts.

In order to isolate the effect of the orientation factor on the exploitation of the fiber strengthening capabilities, a 0.3 orientation factor was imposed and Equation (3) was solved. Figure 7b shows the result. The intrinsic tensile strength of the reinforcement showed less variation against MAPP content. The highest variations were found for the composites with reinforcement contents ranging from 20 to 30 wt %. Higher reinforcement contents returned almost constant intrinsic tensile strengths. Nonetheless, the maximum values showed a higher dependence on the MAPP contents, especially in the aforementioned area. The intrinsic tensile strength returned lower values. This was expected, as a more oriented reinforcement, with the same interphase, will need less intrinsic tensile strength to contribute the same as a less oriented reinforcement.

## 5. Conclusions

Composite materials made of polypropylene reinforced with hemp strand fibers were obtained and tensile was tested. A micromechanics analysis helped in obtaining the strength of the interphase and the intrinsic tensile strength of the reinforcements. A novel representation of the results based on a topographic profile was used to present the results.

The presence of coupling agents showed a high impact on the tensile strength of the composites. A 4 wt % of coupling agent was proposed as correct dosage to maximize the tensile strength of the obtained materials. Coupled materials showed a linear evolution of its tensile strength against reinforcement content. Uncoupled composites showed an asymptotic behavior towards 32 MPa tensile strengths for reinforcement contents superior to 20 wt %.

The contribution of the reinforcements to the tensile strength of the composites increased with the percentage of MAPP up to 6 wt % contents. Further MAPP contents caused a descent of the tensile strength.

The interfacial shear strength or the strength of the interphase between the reinforcements and the matrix was heavily impacted by the MAPP contents and less by the percentage of reinforcement. MAPP contents in the range between 4 and 6 wt % returned the strongest interphases. The strength of the interphase was more sensible under circumstances with a scarcity of the coupling agent rather than when there is an excess of it. A critical zone where the dosage of MAPP affected more noticeably the strength of the interphase was found for 30 wt % reinforcement contents.

The computed intrinsic tensile strength of the reinforcements was affected by its content in the composite. The percentage of coupling agent had little effect of the intrinsic tensile strength of the reinforcement. The presence of 6 wt % of MAPP returned the highest intrinsic tensile strengths, showing an optimal exploitation of the reinforcing capabilities of hemp strands. A local maximum of 625 MPa was found for a composite with 40 wt % reinforcement and 6 wt % MAPP contents.

The representation of the results as a topographic map allowed a fast interpretation of the impact of reinforcement and MAPP contents on the strength of the interphase and the exploitation of the reinforcements.

## Figures and Tables

**Table 1:** Result of the tensile test of hemp strand reinforced polypropylene composites at different coupling agent contents.

Material	*σ_t_^C^* (MPa)	*E_t_^C^* (GPa)	*ε_t_^C^* (%)	σ_t_^m*^ (MPa)
PP	27.6 ± 0.5	1.5 ± 0.1	9.3 ± 0.2	–
20HS0MAPP	29.5 ± 0.7	2.9 ± 0.1	3.4 ± 0.1	24.2
20HS2MAPP	30.7 ± 0.5	2.9 ± 0.1	3.3 ± 0.1	24.0
20HS4MAPP	33.3 ± 0.4	2.9 ± 0.2	3.6 ± 0.2	24.7
20HS6MAPP	34.7 ± 0.4	2.9 ± 0.1	4.0 ± 0.1	25.5
20HS8MAPP	36.3 ± 0.9	3.0 ± 0.1	3.9 ± 0.1	25.3
30HS0MAPP	32.7 ± 0.5	3.9 ± 0.2	3.1 ± 0.1	23.5
30HS2MAPP	37.2 ± 1.0	4.0 ± 0.1	3.1 ± 0.2	23.5
30HS4MAPP	43.4 ± 1.0	3.9 ± 0.1	3.7 ± 0.2	24.9
30HS6MAPP	42.4 ± 1.5	3.9 ± 0.1	3.5 ± 0.3	24.5
30HS8MAPP	39.8 ± 0.9	3.9 ± 0.1	3.4 ± 0.2	24.3
40HS0MAPP	32.8 ± 0.9	5.2 ± 0.3	2.9 ± 0.2	22.9
40HS2MAPP	44.1 ± 1.5	5.2 ± 0.2	3.5 ± 0.2	24.5
40HS4MAPP	48.8 ± 1.9	5.2 ± 0.1	3.5 ± 0.1	24.5
40HS6MAPP	48.7 ± 1.1	5.2 ± 0.1	3.7 ± 0.2	24.9
40HS8MAPP	46.2 ± 2.0	5.2 ± 0.1	3.5 ± 0.2	24.5
50HS0MAPP	31.4 ± 0.6	6.4 ± 0.2	3.3 ± 0.2	24.0
50HS2MAPP	47.3 ± 1.1	6.3 ± 0.1	3.2 ± 0.1	23.7
50HS4MAPP	55.2 ± 1.3	6.4 ± 0.3	3.2 ± 0.2	23.7
50HS6MAPP	54.8 ± 1.6	6.4 ± 0.2	3.3 ± 0.2	24.0
50HS8MAPP	51.7 ± 1.1	6.3 ± 0.1	3.2 ± 0.1	23.7

**Table 2:** Micromechanic properties of the tensile strength of the hemp strand reinforced poplypropylene composites.

Material	*L^F^* (µm)	*χ*_1_	*τ* (MPa)	*σ_t_^F^* (MPa)
20HS0MAPP	669	0.264	9.52	292.6
20HS2MAPP	640	0.269	10.55	341.0
20HS4MAPP	654	0.270	13.88	397.3
20HS6MAPP	647	0.274	14.94	414.3
20HS8MAPP	647	0.286	13.86	475.3
30HS0MAPP	515	0.290	8.10	324.9
30HS2MAPP	483	0.292	10.17	450.7
30HS4MAPP	483	0.301	15.13	523.2
30HS6MAPP	457	0.299	12.88	548.4
30HS8MAPP	457	0.301	9.77	549.6
40HS0MAPP	387	0.287	7.17	328.1
40HS2MAPP	394	0.268	12.25	563.2
40HS4MAPP	419	0.269	14.53	617.8
40HS6MAPP	407	0.269	14.15	635.2
40HS8MAPP	407	0.269	13.76	581.4
50HS0MAPP	452	0.274	5.38	265.7
50HS2MAPP	437	0.293	9.52	469.8
50HS4MAPP	396	0.301	14.37	514.5
50HS6MAPP	371	0.306	13.93	524.1
50HS8MAPP	371	0.295	12.90	487.9
